# Little Divergence Among Mitochondrial Lineages of *Prochilodus* (Teleostei, Characiformes)

**DOI:** 10.3389/fgene.2018.00107

**Published:** 2018-04-04

**Authors:** Bruno F. Melo, Beatriz F. Dorini, Fausto Foresti, Claudio Oliveira

**Affiliations:** ^1^Departamento de Morfologia, Instituto de Biociências, Universidade Estadual Paulista, Botucatu, Brazil; ^2^Department of Vertebrate Zoology, National Museum of Natural History, Smithsonian Institution, Washington, DC, United States

**Keywords:** DNA barcoding, freshwater fishes, gene flow, Neotropics, Prochilodontidae, South America, taxonomy

## Abstract

Evidence that migration prevents population structure among Neotropical characiform fishes has been reported recently but the effects upon species diversification remain unclear. Migratory species of *Prochilodus* have complex species boundaries and intrincate taxonomy representing a good model to address such questions. Here, we analyzed 147 specimens through barcode sequences covering all species of *Prochilodus* across a broad geographic area of South America. Species delimitation and population genetic methods revealed very little genetic divergence among mitochondrial lineages suggesting that extensive gene flow resulted likely from the highly migratory behavior, natural hybridization or recent radiation prevent accumulation of genetic disparity among lineages. Our results clearly delimit eight genetic lineages in which four of them contain a single species and four contain more than one morphologically problematic taxon including a trans-Andean species pair and species of the *P. nigricans* group. Information about biogeographic distribution of haplotypes presented here might contribute to further research on the population genetics and taxonomy of *Prochilodus*.

## Introduction

Fishes of the characiform family Prochilodontidae are widely distributed across Neotropical freshwaters and represent important fishery resources in South America (Ribeiro and Petrere, 1990; Garcia et al., 2009). The migratory behavior allows them to achieve many hundreds of river kilometers to spawn during rainy seasons (Godinho and Kynard, 2006) and, consequently, permits extensive gene flow among distant populations (Sivasundar et al., 2001; Melo et al., 2013). There is substantial evidence that migration of prochilodontids results in high levels of genetic variability and low levels of population structure (Sivasundar et al., 2001; Rueda et al., 2013; Ferreira et al., 2017; Machado et al., 2017). However, there is no empirical study aimed to detect whether long-distance migrations affect genetic diversification at species level in Neotropical freshwater fishes.

Prochilodontids represent a good model to address such questions because much research on population genetics and phylogeography have provided valuable intraspecific genetic information (Sivasundar et al., 2001; Turner et al., 2004; Hatanaka et al., 2006; Carvalho-Costa et al., 2008; Melo et al., 2013; Rueda et al., 2013; Ferreira et al., 2017; Machado et al., 2017; Sales et al., 2018). Furthermore, recent barcoding studies in focal regions (i.e., using endemic species) have generated a robust mitochondrial database for *Prochilodus* (e.g., Carvalho et al., 2011; Rosso et al., 2012; Pereira et al., 2013; Chagas et al., 2015; Díaz et al., 2016) that, if combined, might be useful for species-level comparisons.

Prochilodontidae is represented by three genera (*Ichtyoelephas, Prochilodus*, and *Semaprochilodus*) spanning 21 species (Castro and Vari, 2004). While *Ichthyoelephas* and *Semaprochilodus* have well-stablished taxonomy, except for questions on species boundaries between *S. kneri* and *S. insignis* (Melo et al., 2016a), the taxonomy of *Prochilodus* remains complex. It has 13 morphologically similar species being two endemic to trans-Andean basins of Río Magdalena (*P. magdalenae*) and Lago Maracaibo (*P. reticulatus*), three from the Amazon basin: the widely distributed *P. nigricans* occupying major tributaries of western Amazon in Colombian, Peruvian, Bolivian, and Brazilian eastern rivers flowing northward such as the Madeira, Tapajós, and Tocantins; *P. rubrotaeniatus*, allopatrically distributed through portions of Rio Negro (i.e., Rio Marauiá) and adjacent Guianese rivers such as the Essequibo, Corantijn, and Marowijne river basins; and the less abundant and endangered *P. britskii* from the Rio Apiacás, a tributary of the upper Rio Tapajós. Remaining species are generally endemic to specific drainages: *P. mariae* (Río Orinoco), *P. lineatus* (La Plata and Rio Paraíba do Sul), *P. argenteus* and *P. costatus* (São Francisco), *P. harttii* and *P. vimboides* (Eastern Brazilian drainages from Rio Pardo to Rio Paraíba do Sul), *P. brevis* (coastal rivers of northeastern Brazil), and *P. lacustris* (Río Parnaíba, Northeastern Brazil). Moreover, species distribution of *Prochilodus* has suffered significant alterations due antropogenic introductions in several rivers of eastern and northeastern Brazil (Castro and Vari, 2004).

Some species groups have very subtle morphological differentiation with species being discriminated by ranges and modal meristic values, and by the biogeographic drainage where they are generally endemic (Castro and Vari, 2004). These are *Prochilodus magdalenae*/*P. reticulatus* from Magdalena-Maracaibo, *P. nigricans*/*P. rubrotaeniatus* from Amazon-Guianas-Orinoco, *P. brevis*/*P. lacustris* from northeastern Brazil, and *P. costatus*/*P. lineatus* from São Francisco-La Plata. Furthermore, a recent molecular phylogeny based on six genes revealed non-monophyly of some species, including *P. magdalenae, P. costatus, P. nigricans*, and *P. rubrotaeniatus* (Melo et al., 2016a). This study also revealed a problematic species complex, the *P. nigricans* group that encompasses several specimens of *P. rubrotaeniatus, P. brevis*, and *P. lacustris* interspersed within the *P. nigricans sensu lato*. Although Melo et al. (2016a) used various specimens of *P. nigricans* from distinct biogeographic zones across the Amazon basin, which still remain to be complete, they did not use an extensive sampling for those other problematic species.

Haplotypic variation has been applied to study the diversity of Neotropical characiform fishes as well as used to address systematic questions through the expansion of DNA barcoding projects (e.g., Pereira et al., 2011; Bellafronte et al., 2013; Castro Paz et al., 2014; Benzaquem et al., 2015; Melo et al., 2016b; Ramirez et al., 2017). The majority of barcoding studies have demonstrated high levels of interspecific variation (Melo et al., 2016b; Silva et al., 2016a) while others present a more reduced variation pattern (Pereira et al., 2011; Rossini et al., 2016). Despite a substantial number of population genetic studies applied to species of *Prochilodus* and the natural abundance of those fishes in South American rivers, no genetic study aimed to address species diversity within the genus currently exists.

In this context, barcode sequences of a higher number of specimens from distant regions in association with modern species delimitation methods and haplotype variation analysis are applicable to better determine species delineation within problematic taxa (e.g., Castro Paz et al., 2014; Costa-Silva et al., 2015; Melo et al., 2016b), as in the case of *Prochilodus*. Here, we aim to detect the effects of migration in species diversification, to delimit species of *Prochilodus* using a high taxon sampling and to advance the resolution of the problematic species boundaries within the genus.

## Materials and methods

### Taxon sampling and DNA sequencing

Specimens were collected under a permanent permission number 13843-1 from MMA/IBAMA/SISBIO and subsequently preserved in 95% ethanol. We included 146 specimens spanning all 13 species of *Prochilodus* collected across all South America plus *Semaprochilodus taeniurus* to root the trees (total 147 taxa). We sequenced barcodes for 19 specimens and supplemented the matrix with 127 additional barcodes of *Prochilodus* available at the public genetic databases Genbank (www.ncbi.nlm.nih.gov/) and Barcode of Life Database (BOLD; www.boldsystems.org/). Supplementary Table S1 contains voucher and locality information and accession numbers for databases.

Genomic DNA was extracted from muscle tissues preserved in 95% ethanol with a DNeasy Tissue kit (Qiagen Inc.; http://www.qiagen.com) according to the manufacturer's instructions. We obtained partial sequences of the mitochondrial gene *cytochrome oxidase c subunit I* by amplifying via polymerase chain reaction (PCR) using the primer described in the literature (Melo et al., 2011) and modifying reaction steps as follow: 12.5 μl as a total volume with 9.075 μl of double-distilled water, 1.25 μl 5x buffer, 0.375 μl MgCl_2_ (50 mM), 0.25 μl dNTP mix, 0.25 μl of each primer at 10 μM, 0.05 μl Platinum Taq DNA polymerase enzyme (5 units/μl, Invitrogen; www.invitrogen.com) and 1.0 μl genomic DNA (10–50 ng). The PCR consisted of an initial denaturation (4 min at 95°C) followed by 28–30 cycles of chain denaturation (30 s at 95°C), primer hybridization (30–60 s at 52–54°C), and nucleotide extension (30–60 s at 72°C). After the visualization of the fragments using 1% agarose gel, we performed the sequencing reaction using dye terminators (BigDye™ Terminator v 3.1 Cycle Sequencing Ready Reaction Kit, Applied Biosystems; http://www.appliedbiosystems.com) purified again through ethanol precipitation. We then loaded the samples onto an automatic sequencer ABI 3130-Genetic Analyzer (Applied Biosystems) at the São Paulo State University, Brazil.

### Species delimitation and population genetic analyses

We assembled and edited the newly generated consensus sequences in Geneious 7.1.9 (Kearse et al., 2012) and aligned the whole matrix with Muscle (Edgar, 2004). This matrix contains 147 taxa (146 *Prochilodus* plus one *Semaprochilodus*) and 648 bp. To evaluate the occurrence of substitution saturation, the index of substitution saturation in asymmetrical (Iss.cAsym) and symmetrical (Iss.cSym) topologies were estimated in Dambe 5.3.38 (Xia, 2013). We used PartitionFinder 1.1.0 (Lanfear et al., 2012) to select the best-fit model of nucleotide evolution for our dataset.

Species were previously identified following the most recent and complete taxonomic revision (Castro and Vari, 2004), and lineages were proposed based on subsequent topologies. Most available sequences are from vouchers already identified by the first author (e.g., Melo et al., 2016b) or from previous studies with endemic species (Carvalho et al., 2011; Rosso et al., 2012; Pereira et al., 2013; Díaz et al., 2016). We then generated overall and pairwise values of genetic distance based on Kimura-2-parameters (K2P)+Gamma using Mega 7.0 (Tamura et al., 2013) and a neighbor-joining tree (NJ) with 1,000 replicates of bootstraps using Geneious 7.1.9. We also performed a maximum likelihood (ML) analysis under RAxML HPC-PTHREADS-SSE3 (Stamatakis, 2006) using five random parsimony trees with the GTRGAMMA model (Stamatakis et al., 2008) without rooting and with other parameters at default. We used the autoMRE function to generate pseudoreplicates through MRE-based stopping criteria (Pattengale et al., 2009) that ran a total of 650 replicates. Stopping criteria determine when enough replicates have been generated so that robust bootstraps under ML analysis become computationally practical (Pattengale et al., 2009).

An ultrametric gene tree was generated in a Bayesian inference with Beast 1.8.0 (Drummond et al., 2012) using two independent runs of 50 millions generations sampling trees every 5000th generation. Convergence was indicated by Tracer v1.5 (Rambaut et al., 2014) with estimated sample sizes (ESS) superior to 200. An appropriate number of trees (first 10%) from each run was discarded as burn-in and the MCMC samples was generated using the maximum clade credibility (MCC) topology in TreeAnnotator v1.4.7 (Drummond et al., 2012) and visualized in FigTree v1.4.3.

The general mixed Yule coalescent (GMYC) method (Pons et al., 2006; Fujisawa and Barraclough, 2013) was performed using the ultrametric gene tree estimated with the exponential growth coalescent model (Griffiths and Tavaré, 1994) and the lognormal relaxed clock model (Drummond et al., 2006), which assumes that the rates of molecular evolution are uncorrelated but log-normally distributed among lineages. Species delimitation through GMYC model was conducted using standard parameters [interval = c(0, 10)] and a single threshold that specifies the transition time between to within species branching. Such analysis was conducted with the package *splits* (Species Limits by Threshold Statistics; http://r-forge.r-project.org/projects/splits) in R v.3.0.0 (R Development Core Team, 2013). GMYC appears to be useful for single-locus analysis (Fujisawa and Barraclough, 2013) but depends on the availability of additional data/analyses from independent characters (Esselstyn et al., 2012). Additionally, we used the Bayesian Poisson Tree Processes model (bPTP) (Zhang et al., 2013) in the bPTP webserver (http://species.h-its.org/ptp/) under default parameters. bPTP does not require an ultrametric gene tree and uses, instead, a nexus tree as input file with branch lengths representing the number of nucleotide substitutions (Zhang et al., 2013). We used a nexus MCC tree generated in Beast 1.8.0 (Drummond et al., 2012) as input file and ran 500,000 generations (thinning = 500). We also used a clustering species delimitation analysis through the Automatic Barcode Gap Discovery (ABGD; Puillandre et al., 2012) that automatically defines sequences into hypothetical candidate species based on confidence limits for intraspecific divergence. We used a pairwise distance matrix generated in Mega 7.0 (Kumar et al., 2016) through K2P+G model and 1,000 pseudoreps as input file into the ABGD webserver (wwwabi.snv.jussieu.fr/public/abgd/abgdweb.html) with other parameters left at defaut.

Population genetic analyses were conducted in order to detect levels of genetic variance among haplotypes. We excluded four taxa and excized flanking regions with elevated missing data to properly run those analyses. This reduced matrix contained 143 taxa and 465 bp. Each mitochondrial lineage previously determined by distance and likelihood analyses was treated as a distinct population. We used DnaSP v.5.10.01 (Librado and Rozas, 2009) to obtain the number of polymorphic sites, haplotype number, and nucleotide/haplotype diversity. In Arlequin 3.5.1 (Excoffier and Lischer, 2010), each mitochondrial lineage was set as a single population with the following hypothetic group structuring (group 1 = outgroup; group 2 = lineage 1; group3 = lineage 2; group 4 = lineages 3, 4, and 5; group 5 = lineages 6, 7, and 8) based on the arrangement from ML and Bayesian trees. We ran an analysis of molecular variance (AMOVA; Excoffier et al., 1992) with 1,000 permutations using conventional F-statistics and generated the haplotype network using the median joining analysis (Bandelt et al., 1999) incorporated in PopART 1.7 (Leigh and Bryant, 2015).

## Results

The final matrix contained 147 taxa, 648 bp, and 154 variable sites (23.8%). Nucleotide frequencies were 21.1% adenine, 25.0% citosine, 16.1% guanine, and 26.4% tymine. The newly generated sequences of *Prochilodus* are deposited at GenBank with accession numbers MH068824–MH068842 (Supplementary Table S1). The Iss indexes indicated no saturation in either transitions and transversions in both asymmetrical (Iss.cAsym) and symmetrical (Iss.cSym) topologies. The overall mean of K2P genetic distances without outgroup was 0.025 ± 0.004. Intraspecific genetic variation ranged from zero within the lineage of *P. magdalenae* and *P. reticulatus* to 0.003 within the lineage of *P. costatus* and *P. lineatus*. The lowest pairwise K2P distance was 0.012 ± 0.004 between *P. harttii* and *P. argenteus*. The highest pairwise K2P distance was 0.103 ± 0.016 between *P. vimboides* and *P. mariae*. Fourteen out of 28 pairwise comparisons received values below 0.03. Table 1 shows intraspecific and interspecific genetic distances of each lineage.

**Table 1:** Pairwise K2P genetic distance among distinct lineages of *Prochilodus* (below diagonal) and standard error (above diagonal).

**N**	**Lineage**	**–**	**1**	**2**	**3**	**4**	**5**	**6**	**7**	**8**
–	*Semaprochilodus taeniurus*	**–**	0.019	0.018	0.018	0.016	0.015	0.017	0.017	0.016
1	*P. vimboides*	0.149	**0.003**	0.012	0.016	0.013	0.014	0.015	0.014	0.014
2	*P. magdalenae/P. reticulatus*	0.148	0.077	**0.000**	0.013	0.010	0.010	0.012	0.011	0.011
3	*P. mariae*	0.132	0.103	0.075	**0.001**	0.007	0.007	0.007	0.008	0.007
4	*P. harttii*	0.126	0.084	0.052	0.025	**0.002**	0.004	0.006	0.007	0.006
5	*P. argenteus*	0.114	0.087	0.057	0.025	0.012	**0.002**	0.007	0.007	0.006
6	*P. brevis/P. britskii/P. lacustris/P. nigricans EA/P. rubrotaeniatus WG*	0.134	0.096	0.063	0.029	0.022	0.026	**0.002**	0.005	0.004
7	*P. nigricans WA/P. rubrotaeniatus EG*	0.137	0.091	0.064	0.034	0.028	0.026	0.016	**0.003**	0.005
8	*P. costatus/P. lineatus*	0.129	0.090	0.059	0.029	0.021	0.019	0.013	0.014	**0.003**

**Figure 1:**
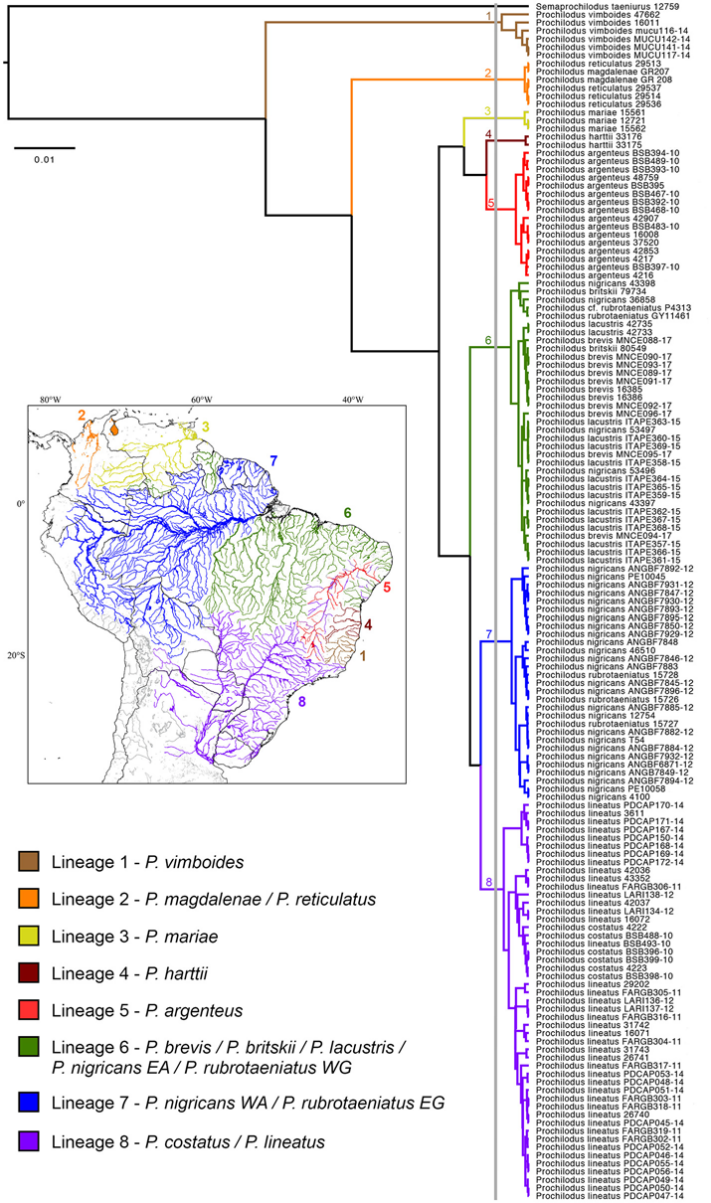
Species delimitation tree based on a Bayesian GMYC analysis showing the single threshold delimiting cladogenetic events at left of the vertical gray bar. Numbers near nodes represent each mitochondrial lineage. All clusters representing species received posterior probabilities = 1. The South American map at left shows the distribution of the genetic lineages of *Prochilodus*.

*Intraspecific genetic distance within lineages in bold. Groups were defined based on the GMYC analysis. N, number of lineage matching those in Figure 1; EA, Eastern Amazon; WA, Western Amazon; EG, Eastern Guianas; WG, Western Guianas*.

Species delimitation analysis by GMYC evidenced the presence of eight genetic lineages (interval 3–22) that encompass the 13 valid species of *Prochilodus* (Figure 1). The threshold time was −0.004 and indicates the time before which all nodes reflect speciation events and after which all nodes reflect coalescent events. Maximum likelihood for the null model was 1452.484 and maximum likelihood for GMYC model was 1457.71. The bPTP species delimitation analysis through both ML and Bayesian approaches returned a slightly distinct result with a total of 10 lineages of *Prochilodus* plus outgroup. The two additional clusters refer to splits within *P. vimboides* (Lineage 1; low support = 0.569) and within *P. lineatus* (Lineage 8; low support = 0.427). ABGD resulted in eight partitions that ranged from 61 (*P* = 0.001) to one candidade species (*P* = 0.03), with one partition with eight candidate species plus outgroup (*P* = 0.002) that match those obtained in GMYC. The evidence of eight species of *Prochilodus* plus outgroup agrees with NJ and ML topologies showing well-defined branches and reciprocal monophyly. Supplementary Figure S1 represents NJ tree and Supplementary Figure S2 represents the best maximum likelihood tree (sum of branch lengths = 0.266).

The reduced matrix for population genetic analyses included 143 sequences (see section Materials and Methods) with 465 bp (364 invariable and 101 polymorphic sites) and a total of 47 haplotypes (H_D_ = 0.951). We found high *F*_ST_ values among lineages/populations (*F*_ST_ = 0.331, *P* < 0.001) ranging from 0.000 (*Prochilodus mariae* vs. *Semaprochilodus, P. harttii* vs. *Semaprochilodus, P. harttii* vs. *P. mariae*) to 1.000 (*P. magdalenae/P. reticulatus* vs. *Semaprochilodus*) but without significant values (Supplementary Table S2). High *F*_ST_ values are expected due the fact that we are treating lineages/species as populations. AMOVA results indicated that there is more variation within populations (66.9%) than among populations within groups (21.7%) or among groups (11.4%) (Supplementary Table S3). The haplotype network shows the distribution and interrelationships among haplotypes (Supplementary Figure S3).

All clusters present strong support for hypothesized lineages in the NJ (bootstrap >74%), ML (bootstrap >76%) and BI (posterior probabilities = 1) analyses. Lineage one includes *Prochilodus vimboides* from eastern Brazil including the Rio Doce, Rio Itaúnas, and Rio Mucuri. Lineage two includes *P. magdalenae* (Río Magdalena in Colombia) and *P. reticulatus* (Lago Maracaibo in Venezuela), the trans-Andean species of *Prochilodus* as a single genetic unit. Third lineage contains three specimens of *P. mariae* from Río Orinoco and lineage four has two specimens of *P. harttii* from Rio Pardo in Eastern Brazil. *Prochilodus argenteus* is represented by lineage five with 14 specimens from the upper, middle and lower Rio São Francisco plus two specimens introduced into Rio Doce and Rio Jequitinhonha. Subsequent lineages (six, seven, and eight) contain more that one species of the *P. nigricans* group (*sensu* Melo et al., 2016a) plus *P. lineatus* and *P. costatus*. The lineage six incorporates the haplotypic group composed by *P. nigricans* from uplands of the Eastern Amazon (Rio Araguaia, upper and middle Rio Tapajós), *P. britskii* from the upper Rio Tapajós (Rio Apiacás), *P. brevis* from northeastern Brazil (states of Ceará and Rio Grande do Norte), *P. lacustris* from Rio Parnaíba, and *P. rubrotaeniatus* from the upper Río Orinoco in Venezuela and the upper Essequibo river basin in Guyana. Lineage seven contains three specimens of *P. rubrotaeniatus* from Corantijn, Coppename, and Marowijne river basins in Suriname plus 26 specimens of *P. nigricans* from lowlands of the Western Amazon, including mainstream Rio Amazonas in Manaus (Brazil), the Río Itaya at the Iquitos region (Peru), Rio Madeira, and Rio Purus. Finally, the eighth lineage contains the species pair composed by *P. costatus* from distinct regions of the Rio São Francisco together with 42 specimens of *P. lineatus* from Rio Paraíba do Sul, upper Rio Paraná, upper Rio Paraguai (all in Brazil) and the lower Rio Paraná (Argentina). Analyses of NJ, ML, and BI returned similar results overall, despite some differences in the arrangement of some lineages (Supplementary Figures S1, S2).

## Discussion

### Species delimitation in *Prochilodus*

Results from the species delimitation analysis revealed the presence of eight genetic lineages covering 13 valid species of *Prochilodus*, in which four lineages (1, 3, 4, and 5) are structured by only one species and the other four lineages (2, 6, 7, and 8) include more than one species. Topologies (Figure 1, Supplementary Figures S1, S2) are quite similar to the molecular phylogeny of Prochilodontidae (Melo et al., 2016a), likely due the locus selection. *Prochilodus vimboides* (lineage 1), for example, splits from the most recent common ancestor of all other *Prochilodus*, although the biogeographic implications for this result still requires a more detailed, time-calibrated analysis of the Prochilodontidae. Other example is the structuring of *P. harttii* (lineage 4) and *P. argenteus* (lineage 5), evidencing distinct genetic lineages even with a recent evidence of hybridization (Sales et al., 2018). In contrast with the molecular phylogeny, results indicate that *P. mariae* is an exclusive cluster, suggesting inconsistencies in the phylogenetic placement of the species (Castro and Vari, 2004; Melo et al., 2016a). A phylogeographic study found that *P. mariae* diverged from *P*. cf. *rubrotaeniatus* in a very recent cladogenesis (Turner et al., 2004), which does not match the Orinoco-Amazon vicariant event resulted from the rise of Vaupes Arch during the Late Miocene (Lujan and Armbruster, 2011).

Our findings suggest the recognition of only one trans-Andean species, in which *Prochilodus magdalenae* from Río Magdalena remains nested within *P. reticulatus* from Lago Maracaibo (lineage 2) as proposed by the molecular phylogeny (Melo et al., 2016a). Species limits among them involve subtle differences in the range and modal values of number of lateral line scales, number of predorsal scales, and number of vertebrae (Castro and Vari, 2004). A further phylogeographic study involving samples from Atrato, Cauca-Magdalena, and Maracaibo might help to elucidate the allopatric distribution of this mitochondrial lineage.

The Amazon basin harbors two distinct mitochondrial lineages of *Prochilodus nigricans* (lineages 6 and 7). A recent study detected population structure in western populations of *P. nigricans* (Madeira and Purus) compared to those from mainstream Rio Amazonas (Machado et al., 2017) despite the lack of samples from eastern tributaries. Interestingly, there is ecological evidence of two distinct migration patterns of *P. nigricans* in the Amazon basin (Araújo-Lima and Ruffino, 2003) that might explain our results. The first involves lateral migrations from floodplain lakes to the mainstream Rio Amazonas with subsequent migration upstream to breeding and spawning (Fernandes, 1997), and the second involves only upstream migrations to upper Rio Tocantins or Araguaia to spawning and downstream migrations to feeding (Carvalho and Mérona, 1986), the latter similar to the well-known pattern observed for *P. argenteus* (Godinho and Kynard, 2006) and *P. lineatus* (Agostinho et al., 2004).

Our results do not support the presence of multiple species within lineage six. *Prochilodus britskii*, a morphologically distinct species, appears for the first time embedded within the lineage, differently from the position as sister to *P. mariae* (Melo et al., 2016a). Both *P. brevis* and *P. lacustris* from northeastern Brazil are distinguished from the species pair *P. nigricans* and *P. rubrotaeniatus* by radial subdivision patterns on body scales (Castro and Vari, 2004). Morphological features diagnosing each of them include overlapped counts of lateral line scales, number of horizontal scale rows below lateral line, and number of circumpeduncular scale rows (Castro and Vari, 2004).

Castro and Vari (2004) redescribed *P. nigricans* by examining almost one thousand Amazonian specimens including type specimens. They designated a neotype from Lago Janauacá at the right margin of Rio Solimões near Manaus in Brazil. Twenty-one individuals of *P. nigricans* from Manaus appear within lineage seven along with specimens from western Amazon. Therefore, this cluster likely constitutes the genetic lineage of the neotype. The position of *P. rubrotaeniatus* still represents a lacuna in our knowledge due the presence of the species in both lineages six and seven. Based on a previous phylogeographic study (Turner et al., 2004), Albert et al. (2011) suggest that *P. rubrotaeniatus* represents an example of paraspecies that gave rise to the endemic *P. mariae*. Paraspecies are paraphyletic, geographically widespread species that originates another peripheral isolated species without becoming extinct (Ackery and Vane-Wright, 1984; Albert et al., 2011). The phylogenetic evidence (Melo et al., 2016a) and the results arrived herein refute such hypothesis and instead, indicate that the present concept of *P. rubrotaeniatus* constitute more than one genetic lineage.

In the Brazilian Shield, *Prochilodus costatus* share the same mitochondrial cluster with *P. lineatus* (lineage 8), which again corroborates the molecular phylogeny (Melo et al., 2016a) and a mitogenome analysis (Chagas et al., 2015). Analyzed specimens of *P. costatus* from Rio Pandeiros/São Francisco are remarkably distant (~2,500 linear km) from analyzed specimens of *P. lineatus* from Rosario in Argentina. These results agree with previous population genetic and phylogeographic studies that show high genetic diversity and low population divergence (Sivasundar et al., 2001; Carvalho-Costa et al., 2008; Melo et al., 2013; Ferreira et al., 2017). A phylogeographic study of *P. lineatus*, for example, found strong similarity among mitochondrial control regions between samples from the lower Rio Paraná in Argentina and upper Rio Paraná in Brazil (Sivasundar et al., 2001). Overlapped counts of lateral line scales, number of vertebrae and allopatry slightly discriminate the two species (Castro and Vari, 2004), which is clearly not supported herein. In addition, our evidence indicates that future population genetic studies of one or another species should include members of both nominal species.

### Little divergence among lineages of *Prochilodus*

Results indicate very little mitochondrial divergence among lineages of *Prochilodus* and provide evidence that distantly sampled specimens, in various instances, correspond to a single mitochondrial lineage. The most plausible hypothesis that might explain such result is that migration affects species diversification. Indeed, migration has been used to explain high levels of gene flow and low population structure in *Prochilodus* (e.g., Sivasundar et al., 2001; Carvalho-Costa et al., 2008; Melo et al., 2013; Ferreira et al., 2017). This is supported by ecological data from fish tagging that found migratory routes of >120 km for *P. argenteus* along the Rio São Francisco (Godinho and Kynard, 2006) and 250 km of *Prochilodus* sp. in that same basin (Paiva and Bastos, 1981).

Migration and gene flow directly influence morphological stasis (Stanley, 1979). This raises some questions about how migration patterns have influenced population diversification without morphological change in *Prochilodus*. Would distinct environmental settings be responsible for distinct movement behaviors along their evolutionary history? López-Fernández and Albert (2011) suggest that massive prochilodontid migrations evolved during the Oligocene, before the separation of the paleo-Amazon-Orinoco river basin, and that posterior vicariant events allowed their successful colonization throughout major Neotropical basins. The two allopatric lineages of *P. nigricans* and their two migration patterns (Carvalho and Mérona, 1986; Fernandes, 1997) support López-Fernández and Albert (2011)'s conclusion and reinforce the fact that a lineage (or population) once fragmented tends to search for ecological adaptation in distinct environmental conditions. Colonization of Neotropical habitats in upland rivers requires adaptation to a strong selective pressure by acquiring specific morphological innovations (Silva et al., 2016b) or behavior specializations, which appears to be the case of *Prochilodus*.

It is noteworthy, however, that migration is not the exclusive factor aging disfavoring species diversification in *Prochilodus*. There are, at least, two more plausible explanations for the observed low genetic variation. Hybridization between the native *P. harttii* and the introduced *P. argenteus* has been genetically identified in the Rio Jequitinhonha recently (Sales et al., 2018). Although hybridization between native species has not been documented yet, the process might be included as another hypothesis to explain our results, at least for sympatric species. Another plausible hypothesis would be recent episodes of species diversification that did not allow accumulation of haplotype variation. Despite a molecular phylogeny is available (Melo et al., 2016a), the lack of a time-calibrated tree does not allow us to support this hypothesis with better confidence. Testing those three most plausible hypotheses to explain the little divergence among lineages of *Prochilodus* is thus a matter of further research.

## Author contributions

BM, FF, and CO designed the project; BM and BD generated, analyzed, and compiled the data; BM wrote most of the text; BM, BD, FF, and CO revised and approved the final version of the manuscript.

### Conflict of interest statement

The authors declare that the research was conducted in the absence of any commercial or financial relationships that could be construed as a potential conflict of interest.

## Supplementary material

Supplementary Table S1 Lineage, taxon, voucher, locality information, and Genbank accession numbers of the analyzed specimens of *Prochilodus*. Lines in bold indicate sequences generated in the present study and asterisks represent BOLD accession numbers.

Supplementary Table S2 Pairwise *F*_ST_ values among mitochondrial lineages of *Prochilodus*. ^*^*P* < 0.05.

Supplementary Table S3 Analysis of molecular variance (AMOVA) among lineages of *Prochilodus*. Groups were ordered on the basis of previous ML and Bayesian analyses (see section Material and Methods).

Supplementary Figure S1 Neighbor-joining tree of the species of *Prochilodus* based on partial sequences of the cytochrome oxidase *c* subunit I. Numbers near nodes represent bootstrap support.

Supplementary Figure S2 Maximum likelihood tree of the *Prochilodus* species based on partial sequences of the cytochrome oxidase *c* subunit I. Numbers near nodes represent bootstrap support. Colors match those in Figure 1.

Supplementary Figure S3 Haplotype network of the eight mitochondrial lineages of *Prochilodus*. Each circle represents a unique haplotype and the size proportional to haplotype frequency. Colors match those in Figure 1.
