# Supplementary material for: Little Divergence Among Mitochondrial Lineages of Prochilodus (Teleostei, Characiformes)
Source: Front Genet. 2018 Apr 4;9:107. doi: 10.3389/fgene.2018.00107 (PMC5893770; doi:10.3389/fgene.2018.00107)
Supplement: Supplementary Table S1 — Lineage, taxon, voucher, locality information, and Genbank accession numbers of the analyzed specimens of Prochilodus. Lines in bold indicate sequences generated in the present study and asterisks represent BOLD accession numbers. [file Table1.DOC]

Supplementary Table S1. Lineage, taxon, voucher, locality information and Genbank accession numbers of the analyzed specimens of *Prochilodus.* Lines in bold indicate sequences generated in the present study and asterisks represent BOLD accession numbers.

| Lin. | Taxon | Voucher | Specimen | Locality | City, State | Country | Genbank or BOLD* numbers |
| --- | --- | --- | --- | --- | --- | --- | --- |
| 1 | *P. vimboides* | LBP 2349 | 16011 | Rio Doce | Sooretama, ES | Brazil | KX086757 |
| 1 | *P. vimboides* | LBP 10180 | 47662 | Rio Mucuri | Carlos Chagas, MG | Brazil | KX086773 |
| 1 | *P. vimboides* | MCNIP 1394 | LGC-4073 | Rio Itaúnas | Mucurici, MG | Brazil | MUCU116-14* |
| 1 | *P. vimboides* | MCNIP 1395 | LGC-4071 | Rio Mucuri | Carlos Chagas, MG | Brazil | MUCU117-14* |
| 1 | *P. vimboides* | MCNIP 1395 | LGC-4041 | Rio Mucuri | Carlos Chagas, MG | Brazil | MUCU141-14* |
| 1 | *P. vimboides* | MCNIP 1395 | LGC-4042 | Rio Mucuri | Carlos Chagas, MG | Brazil | MUCU142-14* |
| 2 | *P. magdalenae* | GR-93-1 | GR207 | Río Magdalena | Magangue, Bolívar | Colombia | KX086779 |
| 2 | *P. magdalenae* | GR-93-1 | GR208 | Río Magdalena | Magangue, Bolívar | Colombia | KX086780 |
| 2 | *P. reticulatus* | LBP 6127 | 29513 | Lago Maracaibo | Encontrados, Zulia | Venezuela | KX086764 |
| 2 | *P. reticulatus* | LBP 6127 | 29514 | Lago Maracaibo | Encontrados, Zulia | Venezuela | KF562435 |
| **2** | ***P. reticulatus*** | **LBP 6127** | **29536** | **Lago Maracaibo** | **Encontrados, Zulia** | **Venezuela** | **MH068824** |
| **2** | ***P. reticulatus*** | **LBP 6127** | **29537** | **Lago Maracaibo** | **Encontrados, Zulia** | **Venezuela** | **MH068825** |
| **3** | ***P. mariae*** | **LBP 1382** | **12721** | **Río Orinoco** | **Caicara del Orinoco, Bolivar** | **Venezuela** | **MH068826** |
| 3 | *P. mariae* | LBP 2188 | 15561 | Río Orinoco | Caicara del Orinoco, Bolivar | Venezuela | KX086755 |
| 3 | *P. mariae* | LBP 2188 | 15562 | Río Orinoco | Caicara del Orinoco, Bolivar | Venezuela | KX086756 |
| 4 | *P. harttii* | LBP 7211 | 33175 | Rio Pardo | Machado Mineiro, MG | Brazil | KX086765 |
| 4 | *P. harttii* | LBP 7211 | 33176 | Rio Pardo | Machado Mineiro, MG | Brazil | KX086766 |
| 5 | *P. argenteus* | LBP 251 | 4216 | Rio São Francisco | Três Marias, MG | Brazil | KX086742 |
| 5 | *P. argenteus* | LBP 251 | 4217 | Rio São Francisco | Três Marias, MG | Brazil | KX086743 |
| **5** | ***P. argenteus*** | **LBP 2349** | **16008** | **Rio Doce** | **Sooretama, ES** | **Brazil** | **MH068831** |
| **5** | ***P. argenteus*** | **LBP 8095** | **37520** | **Rio Jequitinhonha** | **Itapera, BA** | **Brazil** | **MH068828** |
| **5** | ***P. argenteus*** | **LBP 11303** | **42853** | **Rio São Francisco** | **São Roque de Minas, MG** | **Brazil** | **MH068829** |
| **5** | ***P. argenteus*** | **LBP 11303** | **42907** | **Rio São Francisco** | **São Roque de Minas, MG** | **Brazil** | **MH068827** |
| **5** | ***P. argenteus*** | **LBP 11287** | **48759** | **Rio São Francisco** | **Gararu, SE** | **Brazil** | **MH068830** |
| 5 | *P. argenteus* | DCC00250 | BSB392-10 | Rio São Francisco | Urucuia, MG | Brazil | HQ937642 |
| 5 | *P. argenteus* | DCC00253 | BSB393-10 | Rio São Francisco | Urucuia, MG | Brazil | HM405199 |
| 5 | *P. argenteus* | DCC00253 | BSB394-10 | Rio São Francisco | Urucuia, MG | Brazil | HM405200 |
| 5 | *P. argenteus* | DCC00267 | BSB395-10 | Rio São Francisco | Urucuia, MG | Brazil | HM405201 |
| 5 | *P. argenteus* | DCC00290 | BSB397-10 | Rio São Francisco | Urucuia, MG | Brazil | HM405203 |
| 5 | *P. argenteus* | DCC00491 | BSB467-10 | Rio São Francisco | Pandeiros, MG | Brazil | HQ600836 |
| 5 | *P. argenteus* | DCC00490 | BSB468-10 | Rio São Francisco | Pandeiros, MG | Brazil | HQ600835 |
| 5 | *P. argenteus* | DCC00488 | BSB483-10 | Rio São Francisco | Pandeiros, MG | Brazil | HQ600834 |
| 5 | *P. argenteus* | DCC00489 | BSB489-10 | Rio São Francisco | Pandeiros, MG | Brazil | HQ600833 |
| 6 | *P. brevis* | LBP 2496 | 16385 | Açude Araçá, northeastern Brazil | Macaíba, RN | Brazil | KX086759 |
| 6 | *P. brevis* | LBP 2496 | 16386 | Açude Araçá, northeastern Brazil | Macaíba, RN | Brazil | KX086784 |
| 6 | *P. brevis* | UFRN 575 | MNCE088-17 | Piranhas-Açu | Serra Negra do Norte, RN | Brazil | MNCE088-17* |
| 6 | *P. brevis* | UFRN 575 | MNCE089-17 | Piranhas-Açu | Serra Negra do Norte, RN | Brazil | MNCE089-17* |
| 6 | *P. brevis* | UFRN 576 | MNCE090-17 | Piranhas-Açu | Serra Negra do Norte, RN | Brazil | MNCE090-17* |
| 6 | *P. brevis* | UFRN 575 | MNCE091-17 | Piranhas-Açu | Serra Negra do Norte, RN | Brazil | MNCE091-17* |
| 6 | *P. brevis* | UFRN 1835 | MNCE092-17 | Rio Apodi | Apodi, RN | Brazil | MNCE092-17* |
| 6 | *P. brevis* | UFRN 1835 | MNCE093-17 | Rio Apodi | Apodi, RN | Brazil | MNCE093-17* |
| 6 | *P. brevis* | UFRN 594 | MNCE094-17 | Rio Umbuzeiro | Aiuaba, CE | Brazil | MNCE094-17* |
| 6 | *P. brevis* | UFRN 594 | MNCE095-17 | Rio Umbuzeiro | Aiuaba, CE | Brazil | MNCE095-17* |
| 6 | *P. brevis* | UFRN 594 | MNCE096-17 | Rio Umbuzeiro | Aiuaba, CE | Brazil | MNCE096-17* |
| **6** | ***P. britskii*** | **LBP 20260** | **79734** | **Rio Apiacás/Tapajós** | **Alta Floresta, MT** | **Brazil** | **MH068841** |
| **6** | ***P. britskii*** | **LBP 19615** | **80549** | **Rio Apiacás/Tapajós** | **Alta Floresta, MT** | **Brazil** | **MH068842** |
| **6** | ***P. lacustris*** | **LBP 9104** | **42733** | **Rio Poti/Parnaíba** | **Teresina, PI** | **Brazil** | **MH068832** |
| **6** | ***P. lacustris*** | **LBP 9104** | **42735** | **Rio Poti/Parnaíba** | **Teresina, PI** | **Brazil** | **MH068833** |
| 6 | *P. lacustris* | UEMA 104603 | ITAPE357-15 | Rio Itapecuru | Rosário, MA | Brazil | ITAPE357-15* |
| 6 | *P. lacustris* | UEMA 104603 | ITAPE357-15 | Rio Itapecuru | Rosário, MA | Brazil | ITAPE357-15* |
| 6 | *P. lacustris* | UEMA 104603 | ITAPE358-15 | Rio Itapecuru | Rosário, MA | Brazil | ITAPE358-15* |
| 6 | *P. lacustris* | UEMA 104603 | ITAPE359-15 | Rio Itapecuru | Rosário, MA | Brazil | ITAPE359-15* |
| 6 | *P. lacustris* | UEMA 104603 | ITAPE360-15 | Rio Itapecuru | Rosário, MA | Brazil | ITAPE360-15* |
| 6 | *P. lacustris* | UEMA 104603 | ITAPE361-15 | Rio Itapecuru | Rosário, MA | Brazil | ITAPE361-15* |
| 6 | *P. lacustris* | UEMA 104603 | ITAPE362-15 | Rio Itapecuru | Rosário, MA | Brazil | ITAPE362-15* |
| 6 | *P. lacustris* | UEMA 104603 | ITAPE363-15 | Rio Itapecuru | Rosário, MA | Brazil | ITAPE363-15* |
| 6 | *P. lacustris* | UEMA 104603 | ITAPE364-15 | Rio Itapecuru | Rosário, MA | Brazil | ITAPE364-15* |
| 6 | *P. lacustris* | UEMA 104603 | ITAPE365-15 | Rio Itapecuru | Rosário, MA | Brazil | ITAPE365-15* |
| 6 | *P. lacustris* | UEMA 104603 | ITAPE366-15 | Rio Itapecuru | Rosário, MA | Brazil | ITAPE366-15* |
| 6 | *P. lacustris* | UEMA 104603 | ITAPE367-15 | Rio Itapecuru | Rosário, MA | Brazil | ITAPE367-15* |
| 6 | *P. lacustris* | UEMA 104603 | ITAPE368-15 | Rio Itapecuru | Rosário, MA | Brazil | ITAPE368-15* |
| 6 | *P. nigricans* | LBP 7841 | 36858 | Rio Araguaia | Cocalinho, MT | Brazil | KX086767 |
| 6 | *P. nigricans* | LBP 8589 | 43397 | Rio Arinos/Tapajós | Diamantino, MT | Brazil | KX086771 |
| 6 | *P. nigricans* | LBP 8589 | 43398 | Rio Arinos/Tapajós | Diamantino, MT | Brazil | KX086772 |
| 6 | *P. nigricans* | LBP 12865 | 53496 | Rio Tapajós | Itaituba, PA | Brazil | KX086774 |
| **6** | ***P. nigricans*** | **LBP 12865** | **53497** | **Rio Tapajós** | **Itaituba, PA** | **Brazil** | **MH068834** |
| 6 | *P.* cf. *rubrotaeniatus* | ANSP 40692 | P4313 | Río Orinoco | La Esmeralda, Amazonas | Venezuela | KX086784 |
| 6 | *P. rubrotaeniatus* | USNM 403693 | GY11461 | Essequibo river | Cuyuni-Mazaruni | Guyana | KX086782 |
| **7** | ***P. nigricans*** | **LBP 174** | **4100** | **Rio Acre/Purus** | **Rio Branco, AC** | **Brazil** | **MH068838** |
| 7 | *P. nigricans* | LBP 1690 | 12754 | Rio Amazonas | Manaus, AM | Brazil | KX086749 |
| **7** | ***P. nigricans*** | **LBP 10919** | **46510** | **Rio Madeira** | **Porto Velho, RO** | **Brazil** | **MH068839** |
| 7 | *P. nigricans* | OS 18792 | PE10045 | Río Itaya/Amazonas | Maynas, Loreto | Peru | KX086787 |
| 7 | *P. nigricans* | OS 18792 | PE10058 | Río Itaya/Amazonas | Maynas, Loreto | Peru | KX086788 |
| 7 | *P. nigricans* | FMNH 113534 | T54 | Rio Itaya/Amazonas | Iquitos, Loreto | Peru | KX086797 |
| 7 | *P. nigricans* | M3-7 | ANGBF6871-12 | Rio Amazonas | Manaus, AM | Brazil | FJ418758 |
| 7 | *P. nigricans* | H11 | ANGBF7845-12 | Rio Amazonas | Manaus, AM | Brazil | JN032693 |
| 7 | *P. nigricans* | H9 | ANGBF7846-12 | Rio Amazonas | Manaus, AM | Brazil | JN032691 |
| 7 | *P. nigricans* | H7 | ANGBF7847-12 | Rio Amazonas | Manaus, AM | Brazil | JN032689 |
| 7 | *P. nigricans* | H5 | ANGBF7848-12 | Rio Amazonas | Manaus, AM | Brazil | JN032687 |
| 7 | *P. nigricans* | H3 | ANGBF7849-12 | Rio Amazonas | Manaus, AM | Brazil | JN032685 |
| 7 | *P. nigricans* | H1 | ANGBF7850-12 | Rio Amazonas | Manaus, AM | Brazil | JN032683 |
| 7 | *P. nigricans* | Curimata10 | ANGBF7882-12 | Rio Amazonas | Manaus, AM | Brazil | JN007734 |
| 7 | *P. nigricans* | Curimata8 | ANGBF7883-12 | Rio Amazonas | Manaus, AM | Brazil | JN007732 |
| 7 | *P. nigricans* | Curimata6 | ANGBF7884-12 | Rio Amazonas | Manaus, AM | Brazil | JN007730 |
| 7 | *P. nigricans* | Curimata4 | ANGBF7885-12 | Rio Amazonas | Manaus, AM | Brazil | JN007728 |
| 7 | *P. nigricans* | H10 | ANGBF7892-12 | Rio Amazonas | Manaus, AM | Brazil | JN032692 |
| 7 | *P. nigricans* | H8 | ANGBF7893-12 | Rio Amazonas | Manaus, AM | Brazil | JN032690 |
| 7 | *P. nigricans* | H6 | ANGBF7894-12 | Rio Amazonas | Manaus, AM | Brazil | JN032688 |
| 7 | *P. nigricans* | H4 | ANGBF7895-12 | Rio Amazonas | Manaus, AM | Brazil | JN032686 |
| 7 | *P. nigricans* | H2 | ANGBF7896-12 | Rio Amazonas | Manaus, AM | Brazil | JN032684 |
| 7 | *P. nigricans* | Curimata9 | ANGBF7929-12 | Rio Amazonas | Manaus, AM | Brazil | JN007733 |
| 7 | *P. nigricans* | Curimata7 | ANGBF7930-12 | Rio Amazonas | Manaus, AM | Brazil | JN007731 |
| 7 | *P. nigricans* | Curimata5 | ANGBF7931-12 | Rio Amazonas | Manaus, AM | Brazil | JN007729 |
| 7 | *P. nigricans* | Curimata1 | ANGBF7932-12 | Rio Amazonas | Manaus, AM | Brazil | JN007727 |
| 7 | *P. rubrotaeniatus* | MHNG 2705.008 | SU07108 - 15727 | Corantijn river | Kwamalasamutu, Sipaliwini | Suriname | KX086755 |
| 7 | *P. rubrotaeniatus* | MHNG 2717.017 | SU08776 - 15728 | Marowijne river | Palumeu, Sipaliwini | Suriname | KX086766 |
| **7** | ***P. rubrotaeniatus*** | **MHNG 2690.020** | **SU01469 - 15726** | **Coppename river** | **Sipaliwini** | **Suriname** | **MH068840** |
| 8 | *P. costatus* | LBP 252 | 4222 | Rio São Francisco | Três Marias, MG | Brazil | KX086744 |
| 8 | *P. costatus* | LBP 252 | 4223 | Rio São Francisco | Três Marias, MG | Brazil | KX086745 |
| 8 | *P. costatus* | DCC00175 | BSB396-10 | Rio São Francisco | Urucuia, MG | Brazil | HM405202 |
| 8 | *P. costatus* | DCC01095 | BSB398-10 | Rio São Francisco | Buenópolis, MG | Brazil | HM405204 |
| 8 | *P. costatus* | DCC01151 | BSB399-10 | Rio São Francisco | Buenópolis, MG | Brazil | HM405205 |
| 8 | *P. costatus* | DCC00529 | BSB488-10 | Rio São Francisco | Pandeiros, MG | Brazil | HQ600838 |
| 8 | *P. costatus* | DCC00539 | BSB493-10 | Rio São Francisco | Pandeiros, MG | Brazil | HQ600837 |
| 8 | *P. lineatus* | LBP 45 | 3611 | Rio Paraguai | Corumbá, MS | Brazil | KX086741 |
| 8 | *P. lineatus* | LBP 2348 | 16071 | Rio Paraíba do Sul | Campos dos Goytacazes, RJ | Brazil | KX086758 |
| 8 | *P. lineatus* | LBP 2348 | 16072 | Rio Paraíba do Sul | Campos dos Goytacazes, RJ | Brazil | GU702081 |
| 8 | *P. lineatus* | LBP 6250 | 29202 | Upper Rio Paraná | Muzambinho, MG | Brazil | JN989163 |
| 8 | *P. lineatus* | LBP 5189 | 26740 | Upper Rio Paraná | Porto Rico, PR | Brazil | JN989161 |
| 8 | *P. lineatus* | LBP 5189 | 26741 | Upper Rio Paraná | Porto Rico, PR | Brazil | JN989162 |
| 8 | *P. lineatus* | LBP 12651 | 31742 | Upper Rio Paraná | Marilena, PR | Brazil | JN989164 |
| 8 | *P. lineatus* | LBP 12651 | 31743 | Upper Rio Paraná | Marilena, PR | Brazil | JN989165 |
| **8** | ***P. lineatus*** | **LBP 9908** | **42036** | **Rio Paraguai** | **Miranda, MS** | **Brazil** | **MH068835** |
| **8** | ***P. lineatus*** | **LBP 9908** | **42037** | **Rio Paraguai** | **Miranda, MS** | **Brazil** | **MH068836** |
| **8** | ***P. lineatus*** | **LBP 8570** | **43352** | **Rio Paraguai** | **Barra do Bugres, MT** | **Brazil** | **MH068837** |
| 8 | *P. lineatus* | MAG ICT 39 | LARI134-12 | Lower Rio Paraná | Rosario, Santa Fé | Argentina | KU288790 |
| 8 | *P. lineatus* | MAG ICT 41 | LARI136-12 | Lower Rio Paraná | Rosario, Santa Fé | Argentina | KU288792 |
| 8 | *P. lineatus* | MAG ICT 42 | LARI137-12 | Lower Rio Paraná | Rosario, Santa Fé | Argentina | KU288793 |
| 8 | *P. lineatus* | MAG ICT 43 | LARI138-12 | Lower Rio Paraná | Rosario, Santa Fé | Argentina | KU288794 |
| 8 | *P. lineatus* | UNMDP-T 470 | FARGB302-11 | Lower Rio Paraná | Rojas, Buenos Aires | Argentina | JX111831 |
| 8 | *P. lineatus* | UNMDP-T 471 | FARGB303-11 | Lower Rio Paraná | Rojas, Buenos Aires | Argentina | JX111830 |
| 8 | *P. lineatus* | UNMDP-T 472 | FARGB304-11 | Lower Rio Paraná | Rojas, Buenos Aires | Argentina | JX111826 |
| 8 | *P. lineatus* | UNMDP-T 473 | FARGB305-11 | Lower Rio Paraná | Rojas, Buenos Aires | Argentina | JX111828 |
| 8 | *P. lineatus* | UNMDP-T 474 | FARGB306-11 | Lower Rio Paraná | Rojas, Buenos Aires | Argentina | JX111834 |
| 8 | *P. lineatus* | UNMDP-T 484 | FARGB316-11 | Lower Rio Paraná | Rojas, Buenos Aires | Argentina | JX111832 |
| 8 | *P. lineatus* | UNMDP-T 485 | FARGB317-11 | Lower Rio Paraná | Rojas, Buenos Aires | Argentina | JX111829 |
| 8 | *P. lineatus* | UNMDP-T 486 | FARGB318-11 | Lower Rio Paraná | Rojas, Buenos Aires | Argentina | JX111833 |
| 8 | *P. lineatus* | UNMDP-T 487 | FARGB319-11 | Lower Rio Paraná | Rojas, Buenos Aires | Argentina | JX111827 |
| 8 | *P. lineatus* | PDcac131 | PDCAP045-14 | Upper Rio Paraná | Bandeirantes, PR | Brazil | KM897443 |
| 8 | *P. lineatus* | PDcac132 | PDCAP046-14 | Upper Rio Paraná | Bandeirantes, PR | Brazil | KM897457 |
| 8 | *P. lineatus* | PDcac133 | PDCAP047-14 | Upper Rio Paraná | Bandeirantes, PR | Brazil | KM897523 |
| 8 | *P. lineatus* | PDcac134 | PDCAP048-14 | Upper Rio Paraná | Bandeirantes, PR | Brazil | KM897528 |
| 8 | *P. lineatus* | PDcac135 | PDCAP049-14 | Upper Rio Paraná | Bandeirantes, PR | Brazil | KM897315 |
| 8 | *P. lineatus* | PDcac136 | PDCAP050-14 | Upper Rio Paraná | Bandeirantes, PR | Brazil | KM897167 |
| 8 | *P. lineatus* | PDcac137 | PDCAP051-14 | Upper Rio Paraná | Bandeirantes, PR | Brazil | KM897449 |
| 8 | *P. lineatus* | PDcac138 | PDCAP052-14 | Upper Rio Paraná | Bandeirantes, PR | Brazil | KM897351 |
| 8 | *P. lineatus* | PDcac139 | PDCAP053-14 | Upper Rio Paraná | Bandeirantes, PR | Brazil | KM897576 |
| 8 | *P. lineatus* | PDcac140 | PDCAP055-14 | Upper Rio Paraná | Bandeirantes, PR | Brazil | KM897452 |
| 8 | *P. lineatus* | PDcac141 | PDCAP056-14 | Upper Rio Paraná | Bandeirantes, PR | Brazil | KM897216 |
| 8 | *P. lineatus* | PDfcn28 | PDCAP150-14 | Upper Rio Paraná | Bandeirantes, PR | Brazil | KM897381 |
| 8 | *P. lineatus* | PDfcn10 | PDCAP167-14 | Upper Rio Paraná | Bandeirantes, PR | Brazil | KM897316 |
| 8 | *P. lineatus* | PDfcn11 | PDCAP168-14 | Upper Rio Paraná | Bandeirantes, PR | Brazil | KM897671 |
| 8 | *P. lineatus* | PDfcn12 | PDCAP169-14 | Upper Rio Paraná | Bandeirantes, PR | Brazil | KM897644 |
| 8 | *P. lineatus* | PDfcn13 | PDCAP170-14 | Upper Rio Paraná | Bandeirantes, PR | Brazil | KM897300 |
| 8 | *P. lineatus* | PDfcn14 | PDCAP171-14 | Upper Rio Paraná | Bandeirantes, PR | Brazil | KM897253 |
| 8 | *P. lineatus* | PDfcn15 | PDCAP172-14 | Upper Rio Paraná | Bandeirantes, PR | Brazil | KM897204 |
| - | *Semaprochilodus taeniurus* | LBP 1691 | 12759 | Rio Amazonas | Manaus, AM | Brazil | KX086752 |

Supplementary Table S2. Pairwise *F*_ST_ values among mitochondrial lineages of *Prochilodus*. * *P* < 0.05.

| N | Lineage | - | 1 | 2 | 3 | 4 | 5 | 6 | 7 | 8 |
| --- | --- | --- | --- | --- | --- | --- | --- | --- | --- | --- |
| - | *Semaprochilodus taeniurus* | 0.000 |  |  |  |  |  |  |  |  |
| 1 | *P. vimboides* | 0.666 | 0.000 |  |  |  |  |  |  |  |
| 2 | *P. magdalenae / P. reticulatus* | 1.000 | 0.817* | 0.000 |  |  |  |  |  |  |
| 3 | *P. mariae* | 0.000 | 0.510 | 0.772* | 0.000 |  |  |  |  |  |
| 4 | *P. harttii* | 0.000 | 0.510 | 0.772 | 0.000 | 0.000 |  |  |  |  |
| 5 | *P. argenteus* | 0.404 | 0.494* | 0.580* | 0.323 | 0.323 | 0.000 |  |  |  |
| 6 | *P. brevis / P. britskii / P. lacustris / P. nigricans EA / P. rubrotaeniatus WG* | 0.340 | 0.438* | 0.510* | 0.274 | 0.274 | 0.367* | 0.000 |  |  |
| 8 | *P. costatus / P. lineatus* | 0.108 | 0.292* | 0.365* | 0.082 | 0.082 | 0.231* | 0.219* | 0.000* |  |
| 7 | *P. nigricans WA / P. rubrotaeniatus EG* | 0.263 | 0.393* | 0.468* | 0.207 | 0.207 | 0.326* | 0.303* | 0.181* | 0.000 |

Supplementary Table S3. Analysis of molecular variance (AMOVA) among lineages of *Prochilodus*. Groups were ordered on the basis of previous ML and Bayesian analyses (see Material and Methods).

| Source of variation | d.f. | Sum of squares | Variance componentes | % variation |
| --- | --- | --- | --- | --- |
| Among groups | 4 | 8.604 | 0.06133 Va | 11.38 |
| Among populations within groups | 4 | 10.640 | 0.11700 Vb | 21.72 |
| Within populations | 134 | 48.301 | 0.36046 Vc | 66.90 |
