# Supplementary figures and images for: Little Divergence Among Mitochondrial Lineages of Prochilodus (Teleostei, Characiformes)
Source: Front Genet. 2018 Apr 4;9:107. doi: 10.3389/fgene.2018.00107 (PMC5893770; doi:10.3389/fgene.2018.00107)

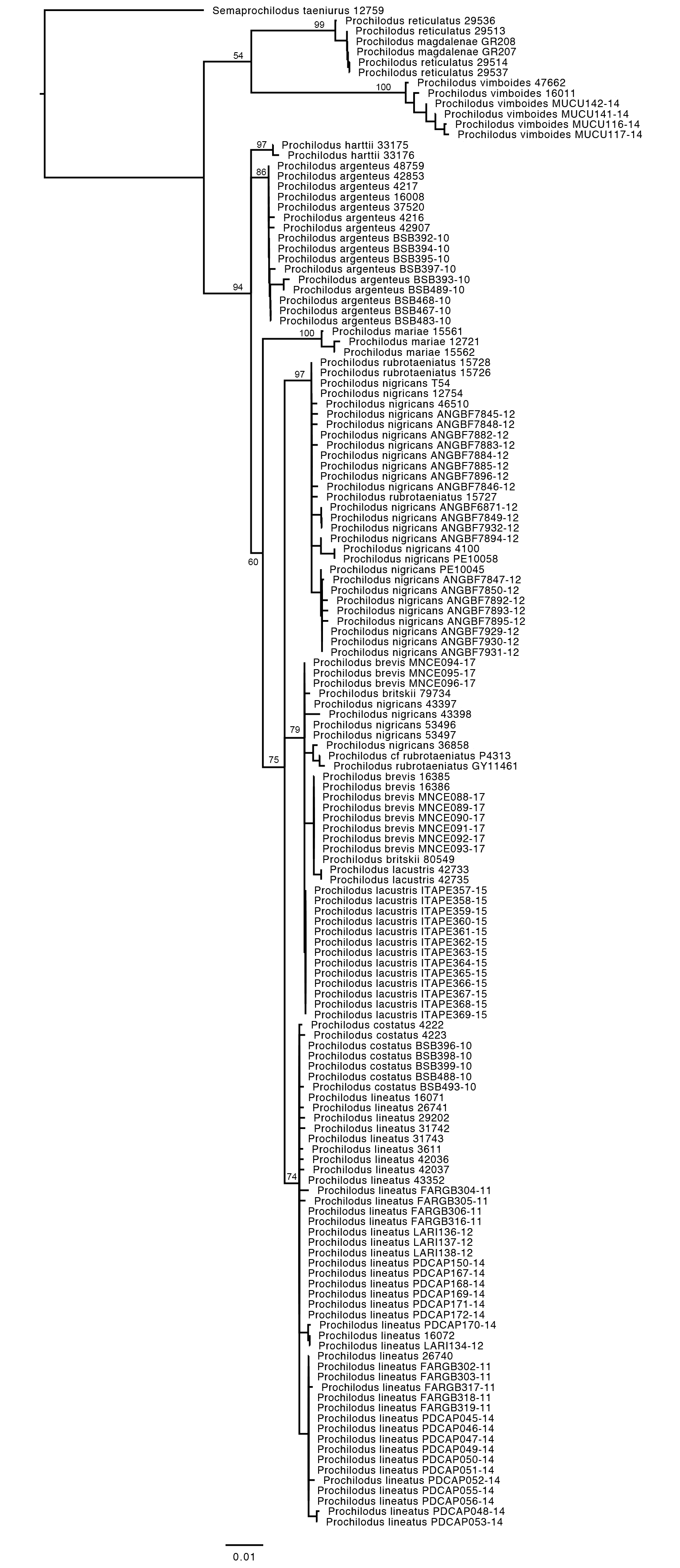

Supplement: Supplementary Figure S1 — Neighbor-joining tree of the species of Prochilodus based on partial sequences of the cytochrome oxidase c subunit I. Numbers near nodes represent bootstrap support. [file Image1.tif]

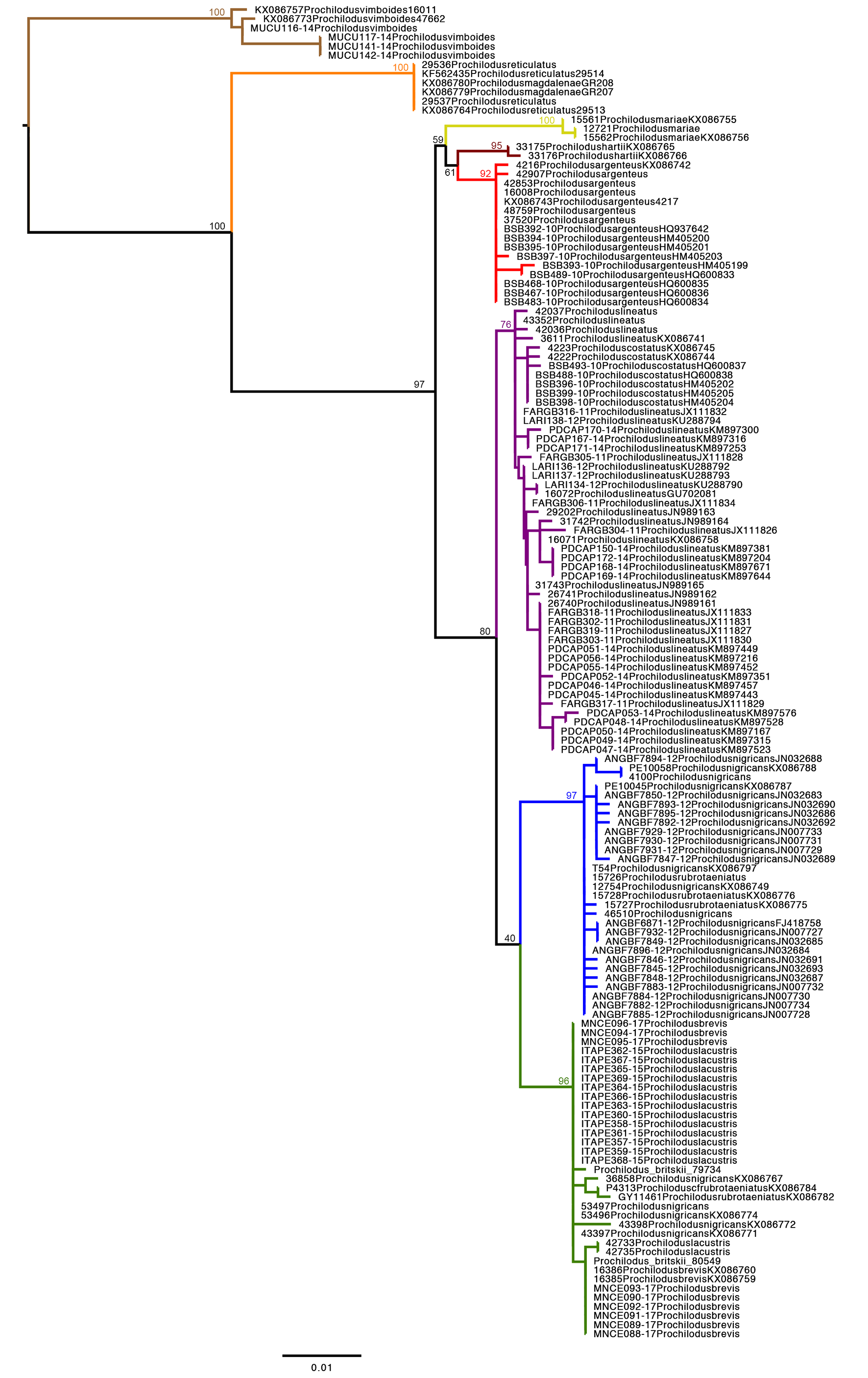

Supplement: Supplementary Figure S2 — Maximum likelihood tree of the Prochilodus species based on partial sequences of the cytochrome oxidase c subunit I. Numbers near nodes represent bootstrap support. Colors match those in Figure 1. [file Image2.tif]

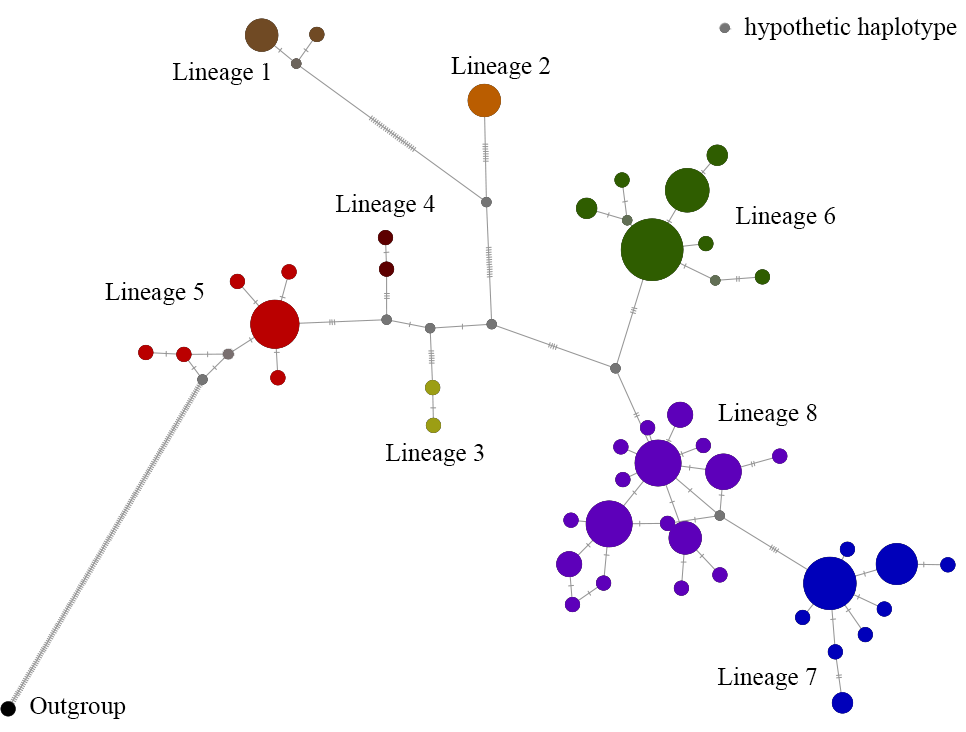

Supplement: Supplementary Figure S3 — Haplotype network of the eight mitochondrial lineages of Prochilodus. Each circle represents a unique haplotype and the size proportional to haplotype frequency. Colors match those in Figure 1. [file Image3.tif]
